# Supplementary material for: Mathematical Contact Tracing Models for the COVID-19 Pandemic: A Systematic Review of the Literature
Source: Healthcare (Basel). 2025 Apr 18;13(8):935. doi: 10.3390/healthcare13080935 (PMC12026787; doi:10.3390/healthcare13080935)
Supplement: Supplementary file 1 [file healthcare-13-00935-s001.zip › healthcare-3550689-supplementary.pdf]

Ocagli et al. 2025 Mathematical contact tracing models for the COVID-19 pan-demic: A systematic review of the literature

Supplementary materials

Sommario

Table S1. Characteristics of the included studies. 2

Table S2. Overview of contact tracing strategies in the included modeling studies, stratified by model type 7

Table S3. Compartments included in SEIR models across the selected studies. Compartments refer to standard transmission stages and contact tracing-specific categories. 15

Table S4. Quality assessment of included studies using the modified Harris et al. tool: individual responses to each criterion and total score. 18

Figure S1 Number of studies by quality level 20

References 21

Table S1. Characteristics of the included studies.

Author, year	N participants	Population	Country	Transmission model structure	Basic reproduction number	Incubation period	Latent period	Infectious period	Quarantine	Isolation	Duration of quarantine (days)	Duration pre-symptomatic period (days)
Almagor 2020 (53)	103000	GP	United Kingdom	Agent based	2.8	2			Yes	Yes		1-3
Amaku 2021 (20)	44639899	GP	Brazil	Compartmental		1.5	1.45		Yes	Yes	14	
Ashcroft 2022 (21)		SD	Switzerland	Branching process	1.76				Yes	Yes		
BahaRaja 2022 (22)	32600000	GP	Malaysia	Compartmental	1.5		5		Yes	Yes		
Biala 2022 (23)		GP	United States	Compartmental					Yes	Yes		
Browne 2022 (24)		GP	China	Compartmental				4.64	Yes	Yes	14	
Chen 2021 (25)	30000 (10000 to 50000)	SD	South Corea	Compartmental	3.6	3	0.5	8.5	Yes	Yes	14	2.5
Chiba 2021 (26)	75614	GP	Japan	Agent based					Yes	Yes	14	
Chiu 2020 (27)		GP	United States	Compartmental					Yes	Yes		
Colomer 2021 (28)	46014554	GP	Spain	Agent based	4	5.1		14	Yes	Yes		

Elias 2022 (54)	47000000, simulation 1000	GP	Spain	Compartmental		5		12	Yes	Yes		
Endo 2021 (62)		SD	United Kingdom	Branching process	1.85				Yes	Yes		
Ferrari 2021 (55)	60589085	GP	Italy	Compartmental		5.1		5.1	Yes	Yes	15	2
Ferretti 2020 (29)		SD		Mathematical		5.5						
Gardner 2021 (30)	100000	SD	United States	Compartmental	1.33; 1.57		3.2		Yes	Yes	14	1
Ge 2021 (456	54000000	GPs	China	Compartmental	3.5		4		Yes	Yes	1-15	2
Getz 2021 (31)	100000-10000000	SD	United States	Compartmental		5	4	7	Yes	Yes		
Gill 2020 (47)	32600000	GP	Malaysia	Compartmental	1.68	6.5		3.6	Yes	Yes	14	
Giordano 2020 (48)	60000000	GP	Italy	Compartmental								
Grantz 2021 (63)		SD	United States	Compartmental	2.5	5.5			Yes	Yes		
Grimm 2021 (79)	83000000	GP	Germany	Compartmental	2.6; 1	5		10 S; 12.5 As	Yes	Yes		
Hellewell 2020 (33)		SD	United Kingdom	Branching process	1.5; 2.5; 3.5	5.8						
Hernandez-Orallo 2020 (34)	1000000	SD	Spain	Compartmental	3						14	

Hinch 2021 (56)	1000000	GP	United Kingdom	Agent based					Yes	Yes	14
Hoops 2021 (57)		GP	United States	Agent based					Yes	Yes	
Hornstein 2022 (35)		SD	United States	Compartmental		6	3	8	Yes	Yes	
Hu 2021 (64)	3000	SD	China	Compartmental	2.2	5	6.4		Yes	Yes	
Humphrey 2021 (49)		GP	South Corea, Canada, Italy, United States	Compartmental	2.38; 2.1; 2.11; 3.07				Yes	Yes	
James 2021 (52)	101	GP	New Zealand	Branching process					Yes	Yes	
Kerr 2021 (36)	2250000	SD	United States	Agent based	2.5				Yes	Yes	14
Khajanchi 2021 (65)	India (160000); Kerala (40000); Andhra Pradesh (130000); West Bengal (120000); Maharashtra (1000000); Karnataka (200000).	SD	India	Compartmental	1.6795; 3.3870; 1.8155; 1.5525; 1.2937; 1.5934	8					

Kim 2021 (43)	20000	SD	South Corea	Agent based	2.5	5.5		8			
Kretzschmar 2020 (44)	1000	SD		Branching process	2.5		1-3	10			
Kucharski 2020 (37)	40162	GP	United Kingdom	Agent based	2.6	5		5			
Lanzarotti 2021 (38)	2000	SD		Agent based			1-3				
Maiorana 2021 (50)		SD		Mathematical							
Mancastroppa 2021 (45)		SD		Activity-driver network							1.5
McQuade 2021 (58)	“red zone” (4111309); “orange zone” (2835111); “yellow zone” (1845474)	GP	United States	Compartmental	4		4-14	6	Yes	14	
Mettler 2021 (66)	1000	SD		Agent based	1.93	1.63			No		
Pollmann 2021 (39)	10000, 100000, 1000000	SD		2 compartmental; 2 agent based model	2.0;3.0;4.0	(0, 3.06, 2.44) , (0, 3.06, 1.3)			Yes	14	
Rajabi 2021 (67)	5000	SD		Agent based	1.95	5			Yes		
Ramos 2021 (59)	60317000	GP		Compartmental				14	Yes	14.2729	5.5

Rusu 2021 (68)	200, 500, 1000, 2000, 5000, 10000,	SD		Compartmental	3.18		3.7	2.3			1.5
Ryu 2021 (40)	10000	SD	South Corea	Agent based	2.84	5		14	Yes		
Sasmita 2020 (51)	268000000	GP	Indonesia	Compartmental	2.25						
Scarabel 2021 (69)		SD		Mathematical	2.5; 1.5	4.84		20	No		
Shayak 2021 (70)	302400	SD		Cst		5		3			
Soldano 2021 (71)	100000	SD		Agent based					No	No	
Sturniolo 2021 (72)	67000000	SD		Agent based	3	5					
Tatapudi 2020 (41)	2800000	GP	United States	Agent based		5.5	3	9.5			
Traore 2020 (60)	11081000	GP		Compartmental		7					
Wang 2020 (42)	32583	GP	China	Compartmental					Yes	No	

Abbreviations: As = Asymptomatic; CT = contact tracing; GP = general population; SD = simulated data; S = symptomatic

Table S2. Overview of contact tracing strategies in the included modeling studies, stratified by model type

Author year	Digital CT	Different scenarios	New compartments used	Assumptions	Type of CT
**Compartmental**					
Amaku 2021 (20)				CT only for S	
Baharaja 2022 (22)			E: E not traced;<br>T: I and E and T and<br>Q: Q (S, As)		
Biala 2022 (23)		CT activity			
Browne 2022 (24)			N, T, self-Q		
Chen 2021 (25)		Different % of CT activity			
Chiu 2020 (27)			A: infectious and asymptomatic;<br>F: dead		
Elias 2022 (54)		No Q, forward CT, intermediate backward CT and complete backward CT	Q: Q individuals		Backward and forward
Ferrari 2021 (55)	App	Different proportion of users	Q and P		

Gardner 2021 (30)				Different coefficients
Ge 2021 (46)		Different % of CT activity	Qp: preclinical symptomatic cases in quarantine;<br>Qc: clinical symptomatic cases in quarantine;<br>P: preclinical symptomatic cases;<br>C: clinical symptomatic cases;	E: proportion p is quarantined (thanks to CT); 1-p is lost and isolated later
Getz 2021 (31)	Web app		C: contact class;<br>L: latent class	
Gill 2020 (47)			T: traced close contact and a negative test result population;<br>Eq: traced exposed close-contact and positive test result population<br>Undergoing quarantine;<br>Iq: infected isolated	

Giordano 2020 (48)			T: threatened (infected with life-threatening symptoms,<br>Detected);<br>D (diagnosed (asymptomatic infected, detected);<br>A: ailing (symptomatic infected, undetected);<br>R: recognized (symptomatic infected, detected)		
Grantz 2021 (63)		Basic model framework, to which real-world complexity is then added	D, Q, undetected in Community		
Grimm 2021 (32)	App		Inside E		
Hernandez-orallo 2020 (34)	App		Q		Forward
Hornstein 2022 (35)		More effective Q, CT, random testing	Inside E and I	S and As	
Hu 2021 (64)		Testing and tracing			

Humphrey 2021 (49)			L: isolated people	
Khajanchi 2021 (65)			Isolated and hospitalised	
Mcquade 2021 (58)			Inside S, E, I, R	
Ramos 2021 (59)			New coefficients and new compartments inside I and R	
Rusu 2021 (68)	App		I and traced individuals	
Sasmita 2020 (51)		3 scenarios depending on the controls	I1 (carrier),<br>I2 (infectious)	5 strategies for control: large-scale social restriction, contact tracing, mass testing, case detection and treatment, and the wearing of face masks
Traore 2020 (60)				E, Q and untraced;<br>Proportion q of exposed individuals is quarantined; 1-q is untraced
Wang 2020 (42)			New coefficients (Q, C)	
**Agent based**				

Almagor 2020 (53)	App			Positive has to notify; contacts of previous 10 days are traced	
Chiba 2021 (26)	App			Q and not	
Colomer 2021 (28)		Different % of CT activity			
Hinch 2021 (56)	App	Real-world-imperfections affecting CT programmes and testing		It models recursive contact tracing with and without testing	
Hoops 2021 (57)		No CT, baseline, more CT		Various interventions: NPI and vaccine allocation	
Kerr 2021 (36)		Different % of CT activity			Backward and forward
Kim 2021 (43)					
Kucharski 2020 (37)	App	Different % of CT activity			
Lanzarotti 2021 (38)				Dynamics of CT	
Mettler 2021 (66)		CT/not CT			
Rajabi 2021 (67)					
Ryu 2021 (40)		Including CT and isolation		Model uses a contact nework	

Soldano 2021 (71)	App	Mct + dct (app)	Mct applied to all detected cases: 40% of contacts are assumed to be traced and isolated	
Sturniolo 2021 (72)			Every contact is traced; individuals move through compartments	
Tatapudi 2020 (41)			Uses parameter to reflect restrictions, CT	
**Branching process**				
Ashcroft 2022 (21)			CT for symptomatics; a fraction of secondary contacts is assumed to be quarantined	Forward
Endo 2021 (62)		Backward CT, backward+forward CT		Backward and forward
Hellewell 2020 (33)		Simulation at different p	Contact is traced with probability p	
James 2021 (52)			Parameters: proportion of traced contacts; Mean time to trace contacts following a positive test;	Backward and forward

Kretzschmar 2020 (44)	App			Effectiveness of contact Q and case isolation	
			**Other models**		
Ferretti 2020 (29)	App			Reduction of R0 depends on proportion of I and proportion of Q contacts	
Maiorana 2021 (50)	App				Forward
Mancastroppa 2021 (45)	Mct and dct	Two CT protocols (in the same conditions)		Parameters turned to values derived from empirical observations	Backward and forward
Pollmann 2021 (39)	Dct	Effect of tracing delays		Dct combined with random testing and social distancing	Backward and forward
Scarabel 2021 (69)		Short-term CT interruption		Cases tested S and tracing	Forward
Shayak 2021 (70)				Human interaction is heterogeneous; S probability P1, missed probability 1-P1	Forward

Abbreviations: As = Asymptomatic; CT = Contact tracing; dct = digital contact tracing; E = Exposed; I = Infected; L = Latent; mct = manual contact tracing; N = Normal; Q = quarantind; S = Symptomatic; T = Traced

Table S3. Compartments included in SEIR models across the selected studies. Compartments refer to standard transmission stages and contact tracing-specific categories.

Author year	Susceptible	Exposed	Infected/infective	Recovered	Death	Hospitalized	Asymptomatic infected	Quarantine	Traced	Other
Amaku 2021 (20)	N, I-Su, Su previously I	Yes	Yes	Yes		Yes	Yes	Yes		Gt: severe disease ICU
BahaRaja 2022 (22)	Yes	I in un-Test	S, As	with immunity	S, As			S, As	Yes	V: vaccinated
Biala 2022 (23)	Yes	T, nonT	T, monitored/S	Yes	Yes	Yes				
Browne 2022 (24)	N, T, self-Q	N, T, self-Q	N, T, self-Q	Yes						
Chen 2021 (25)	Yes	Yes	S: infectious and S superspreaders, infectious and S non-superspreaders	Yes		Yes	As superspreaders, As non-superspreaders	XQ: Q uninfected contacts,<br><br>Qr: I/Q infected or mild illness or remain As; Qh, I/Q infected severe/critical illnesses		
Chiu 2020 (27)	N, T	N, T	non-Test infected and I,<br><br>T infected and Test infected	Test, non-Test	Yes		non-Test As,<br><br>T As		I: isolated,<br><br>T: infected	
Elias 2022 (54)	Yes	Yes	Yes	Yes	Yes			Yes		

Ferrari 2021 (55)	Yes	Yes	Yes	Yes			Yes	Su, infected, As and pre-S		P: pre-S infected
Gardner 2021 (30)	Yes	Yes	Yes	Yes				Yes		
Ge 2021 (46)	Yes	Yes		Yes				preclinical and clinical symptom		P: pre-S infected; C: S infected
Getz 2021 (31)	Yes	contact and latent	S		Yes		Yes			V: immune
Gill 2020 (47)	Yes	N, T close-contact	N, isolated	Yes					T: traced close-C and negative test	
Giordano 2020 (48)	Yes		As non Test	Yes	Yes				T: critical infected and Test	D diagnosed: As Test; A: S non-Test; R: S Test
Grantz 2021 (63)			Test and I, infections among Q C of cases, undetected							
Grimm 2021 (32)	Yes	Yes	As, S, severe symptom	Yes	Yes					
Hernandez-Orallo 2020 (34)	Yes		Yes	Yes				T infected, Su T, T and Test infected		
Hornstein 2022 (35)	Yes	N, T	S, As, As T	Yes	Yes					

Hu 2021 (64)	Yes	Yes	infected, As	Yes	confirmed					Is: infected S; T: test-positive who are Q; f: deaths no positive test confirmation
Humphrey 2021 (49)	Yes	Yes	Yes	Yes						L: I
Khajanchi 2021 (65)	Yes	Yes	S	Yes		Yes	Yes			
McQuade 2021 (58)	N, d	N, Q	S, As, Q	Yes	Yes					
Ramos 2021 (59)	Yes	Yes	N, undetected, undetected, death	detected, undetected	known and not	N, deaths				
Rusu 2021 (68)	Yes	Yes	N, S, As	Yes		Yes			T: T and I	
Sasmita 2020 (51)	Yes	Yes	detected, undetected	Yes						S: Su (not more immune)
Traore 2020 (60)										
Wang 2020 (42)	Yes	latent infected	Yes	Yes	Yes	Yes		Q: infected and not		C: close C (not infected)

As = Asymptomatic; C = Contact; I = Isolated; ICU = Intensive Care Unit; N = Normal; S = Symptomatic; Su = susceptible; T = Traced; Test = Tested; Q = Quarantine

Ocagli et al. 2025 Mathematical contact tracing models for the COVID-19 pan-demic: A systematic review of the literature

T

Author, Year	Q1	Q2	Q3	Q4	Q5	Q6	Q7	Q8	Q9	Q10	Q11	Q12	Q13	Q14	Quality level
Almagor 2020	Yes	Yes	Yes	Partial	Partial	Yes	Yes	Yes	No	Yes	No	Yes	Yes	Yes	High
Amaku 2021	Yes	Yes	Partial	Partial	Yes	Yes	Yes	Yes	Yes	Yes	No	Partial	Yes	Yes	Very High
Ashcroft 2022	Yes	Partial	Yes	Yes	Yes	Yes	Yes	Yes	Partial	Partial	Partial	Partial	Yes	Yes	Very High
BahaRaja 2022	Yes	Partial	Partial	Yes	Yes	Yes	Partial	Yes	Yes	Yes	No	Partial	Yes	Yes	High
Biala 2022	Yes	Partial	Yes	Yes	Yes	Yes	Yes	Partial	No	Yes	No	Partial	Yes	No	High
Browne 2022	Yes	Partial	Yes	Partial	Partial	Partial	Partial	Partial	Yes	Yes	No	Yes	Partial	Yes	High
Chen 2021	Yes	Yes	Yes	Partial	Yes	Partial	Yes	Yes	Yes	Yes	No	Yes	Yes	Yes	Very High
Chiba 2021	Yes	Partial	Partial	Partial	Yes	Partial	Partial	Partial	Yes	Partial	Partial	Partial	Partial	Yes	Medium
Chiu 2020	Yes	Partial	Partial	Yes	Yes	Yes	Yes	Partial	Partial	Yes	Yes	Partial	Yes	Partial	High
Colomer 2021	Yes	Partial	Partial	Yes	Partial	Partial	Partial	Partial	Partial	Partial	No	Yes	Yes	Yes	Medium
Elias 2022	Yes	Partial	Partial	Partial	Partial	Yes	Partial	Yes	Partial	Partial	Partial	Partial	Yes	Partial	Medium
Endo 2021	Partial	Partial	Partial	Partial	Partial	Partial	Partial	Partial	Partial	Partial	Partial	Partial	Partial	Partial	Medium
Ferrari 2021	Yes	Yes	Partial	Partial	Yes	Partial	Partial	Partial	Yes	Partial	Partial	Partial	Yes	Partial	High
Ferretti 2020	Yes	Partial	Partial	Yes	Partial	Partial	Yes	Partial	Partial	Partial	No	Partial	Yes	Partial	Medium
Gardner 2021	Yes	Partial	Yes	Yes	Partial	Partial	Partial	Partial	Partial	Partial	No	Yes	Partial	Partial	Medium
Ge 2021	Yes	Yes	Partial	Partial	Partial	Partial	Yes	Partial	Partial	Partial	Partial	Partial	Yes	Partial	Medium
Getz 2021	Yes	Partial	Partial	Partial	Partial	Partial	Partial	Partial	No	Partial	No	Partial	Yes	Yes	Medium
Gill 2020	Yes	Yes	Partial	Partial	Yes	Yes	Partial	Partial	No	Partial	No	Partial	Yes	Yes	Medium
Giordano 2020	Partial	Partial	Partial	Partial	Partial	Yes	Partial	Partial	Yes	Yes	No	Partial	No	Partial	Medium
Grantz 2021	Yes	Partial	Yes	Yes	Yes	Partial	Partial	Partial	Partial	Partial	No	Partial	Partial	Partial	Medium
Grimm 2021	Yes	Partial	Yes	Yes	Yes	Yes	Yes	Yes	No	Yes	No	Partial	Partial	Partial	High
Hellewell 2020	Yes	Partial	Yes	Yes	Partial	Yes	Partial	Partial	No	Partial	No	Partial	Partial	Yes	Medium
Hernandez-Orallo 2020	Yes	Partial	Partial	Partial	Partial	Yes	Partial	Partial	No	Partial	No	Partial	Partial	Partial	Medium
Hinch 2021	Yes	Yes	Yes	Yes	Yes	Yes	Partial	Partial	Partial	Yes	Yes	Yes	Yes	Yes	Very High
Hoops 2021	Partial	Yes	Partial	Partial	Partial	Partial	Partial	Partial	No	Partial	No	Partial	Partial	Partial	Low
Hornstein 2022	Partial	Partial	Partial	Partial	Yes	Yes	Partial	Partial	No	Partial	No	Partial	Partial	No	Low
Hu 2021	Yes	Yes	Yes	Yes	Yes	Yes	Partial	Partial	Yes	Yes	No	Yes	Yes	Yes	Very High
Humphrey 2021	Yes	Yes	Partial	Yes	Yes	Yes	Partial	Partial	Partial	Yes	No	Partial	Yes	Partial	High
James 2021	Partial	Yes	Partial	Yes	Partial	Partial	Partial	Partial	Yes	Partial	No	Partial	Yes	Yes	Medium

Ocagli et al. 2025 Mathematical contact tracing models for the COVID-19 pan-demic: A systematic review of the literature

Kerr 2021	Yes	Yes	Yes	Yes	Yes	Yes	Yes	Partial	Yes	Yes	Yes	Yes	Partial	Yes	Very High
Khajanchi 2021	Yes	Yes	Yes	Yes	Yes	Yes	Yes	Partial	Yes	Yes	No	Yes	Partial	Partial	Very High
Kim 2021	Yes	Partial	Yes	Yes	Yes	Partial	Yes	Partial	Yes	No	No	Yes	Partial	Yes	High
Kretzschmar 2020	Yes	Partial	Yes	Yes	Yes	Partial	Yes	Partial	Yes	No	No	Yes	Yes	Yes	High
Kucharski 2020	Yes	Yes	Yes	Yes	Yes	Partial	Yes	Partial	Yes	No	No	Yes	Yes	Yes	High
Lanzarotti 2021	Yes	Partial	Yes	Yes	Yes	Partial	Yes	Partial	No	Partial	No	Partial	Partial	No	Medium
Maiorana 2021	Yes	No	Partial	Yes	Yes	Yes	Partial	Yes	Yes	No	No	Partial	Yes	Yes	High
Mancastroppa 2021	Yes	Partial	Yes	Yes	Yes	Partial	Yes	Partial	No	No	No	Partial	Yes	Partial	Medium
McQuade 2021	Yes	Yes	Yes	Yes	Yes	Yes	Partial	Partial	Yes	Yes	No	Partial	Partial	Partial	High
Mettler 2021	Yes	Partial	Partial	Yes	Yes	Yes	Yes	Partial	Partial	No	No	Partial	Yes	Yes	High
Pollmann 2021	Yes	Partial	Yes	Yes	Yes	Yes	Yes	Partial	Partial	No	Partial	Partial	Yes	Yes	High
Rajabi 2021	Yes	Partial	Yes	Yes	Yes	Partial	No	Partial	No	No	No	Partial	Partial	Yes	Medium
Ramos 2021	Yes	Yes	Yes	Yes	Yes	Yes	Yes	Partial	Partial	Yes	Yes	Partial	Yes	Yes	Very High
Rusu 2021	Yes	Partial	Yes	Yes	Yes	Yes	Partial	Partial	Partial	Yes	Yes	Partial	Partial	Yes	High
Ryu 2021	Yes	Partial	Yes	Yes	Yes	Partial	Partial	Partial	Partial	Partial	No	Yes	Yes	No	Medium
Sasmita 2020	Yes	Partial	Yes	Yes	Yes	Partial	Partial	Partial	No	No	No	Partial	Yes	Yes	Medium
Scarabel 2021	Yes	Partial	Yes	Partial	Yes	Partial	Partial	Partial	Partial	No	No	Partial	Partial	Yes	Medium
Shayak 2021	Partial	Partial	Partial	Partial	Yes	Partial	Partial	Partial	Partial	Partial	No	Partial	Yes	No	Medium
Soldano 2021	Yes	Partial	Partial	Partial	Partial	Partial	Partial	Partial	Partial	Yes	Yes	Yes	Yes	Yes	High
Sturniolo 2021	Partial	Partial	Yes	Partial	Yes	Partial	Yes	Partial	Partial	Partial	No	Yes	Yes	No	Medium
Tatapudi 2020	Partial	Yes	Partial	Yes	Yes	Yes	Partial	Partial	Partial	Partial	Yes	Yes	Yes	Yes	High
Traore 2020	Yes	Partial	Partial	Yes	Yes	Yes	Partial	Partial	No	Partial	No	Partial	Partial	Yes	Medium
Wang 2020	Yes	Yes	Yes	Yes	Yes	Partial	Yes	Partial	Yes	Yes	Yes	Yes	Partial	Partial	Very High

Q1: Aim and objectives; Q2: Setting and population; Q3: Intervention comparators; Q4: Outcome measures; Q5: Model structure and time horizon; Q6: Modeling methods; Q7: Parameters ranges and data sources; Q8: Assumptions explicit and justified; Q9: Quality of data and uncertainty ad or sensitivity analysis; Q10: Method of fitting; Q11: Model validation ; Q12: Presentation of results and uncertainty; Q13: Interpetation and_discussion of results; Q14: Funding source and conflicts of interest.

Figures

Figure S1 Number of studies by quality level

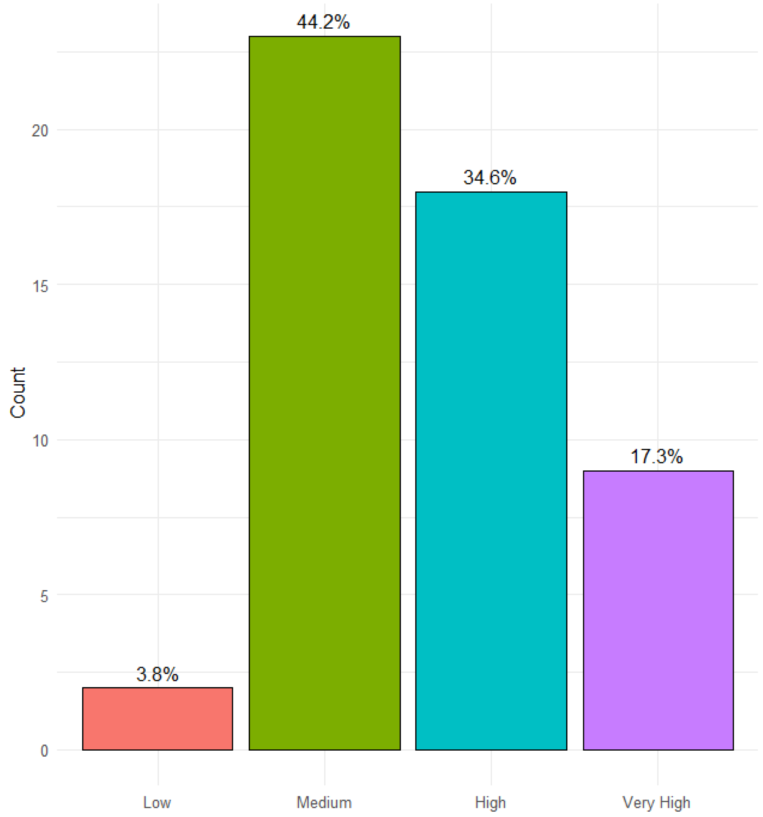

References

1. Almagor, J.; Picascia, S. Exploring the Effectiveness of a COVID-19 Contact Tracing App Using an Agent-Based Model. *Sci. Rep.* **2020**, *10*, doi:10.1038/s41598-020-79000-y.
2. Amaku, M.; Covas, D.T.; Bezerra Coutinho, F.A.; Azevedo, R.S.; Massad, E. Modelling the Impact of Contact Tracing of Symptomatic Individuals on the COVID-19 Epidemic. *Clinics* **2021**, *76*, doi:10.6061/CLINICS/2021/E2639.
3. Ashcroft, P.; Lehtinen, S.; Bonhoeffer, S. Test-Trace-Isolate-Quarantine (TTIQ) Intervention Strategies after Symptomatic COVID-19 Case Identification. *PLoS ONE* **2022**, *17*, doi:10.1371/journal.pone.0263597.
4. Baha Raja D.; Abdul Taib N.A.; Teo A.K.J.; Jayaraj V.J.; Ting C.-Y. Vaccines Alone Are No Silver Bullets: A Modeling Study on the Impact of Efficient Contact Tracing on COVID-19 Infection and Transmission in Malaysia. *Int. Health* **2022**, doi:10.1093/inthealth/ihac005.
5. Biala, T.A.; Afolabi, Y.O.; Khaliq, A.Q.M. How Efficient Is Contact Tracing in Mitigating the Spread of COVID-19? A Mathematical Modeling Approach. *Appl. Math. Model.* **2022**, *103*, 714–730, doi:10.1016/j.apm.2021.11.011.
6. Browne, C.J.; Gulbudak, H.; Macdonald, J.C. Differential Impacts of Contact Tracing and Lockdowns on Outbreak Size in COVID-19 Model Applied to China. *J. Theor. Biol.* **2022**, *532*, doi:10.1016/j.jtbi.2021.110919.
7. Chen, Y.-H.; Fang, C.-T.; Huang, Y.-L. Effect of Non-Lockdown Social Distancing and Testing-Contact Tracing During a COVID-19 Outbreak in Daegu, South Korea, February to April 2020: A Modeling Study. *Int. J. Infect. Dis.* **2021**, *110*, 213–221, doi:10.1016/j.ijid.2021.07.058.
8. Chiba, A. Modeling the Effects of Contact-Tracing Apps on the Spread of the Coronavirus Disease: Mechanisms, Conditions, and Efficiency. *PLoS ONE* **2021**, *16*, doi:10.1371/journal.pone.0256151.
9. Chiu, W.A.; Fischer, R.; Ndeffo-Mbah, M.L. State-Level Needs for Social Distancing and Contact Tracing to Contain COVID-19 in the United States. *Nat. Hum. Behav.* **2020**, *4*, 1080–1090, doi:10.1038/s41562-020-00969-7.
10. Colomer, M.À.; Margalida, A.; Alòs, F.; Oliva-Vidal, P.; Vilella, A.; Fraile, L. Modeling of Vaccination and Contact Tracing as Tools to Control the COVID-19 Outbreak in Spain. *Vaccines* **2021**, *9*, 386, doi:10.3390/vaccines9040386.
11. Elías, L.L.; Elías, S.L.; del Rey, A.M. An Analysis of Contact Tracing Protocol in an Over-Dispersed SEIQR Covid-like Disease. *Phys. Stat. Mech. Its Appl.* **2022**, *590*, doi:10.1016/j.physa.2021.126754.
12. Endo, A.; Leclerc, Q.J.; Knight, G.M.; Medley, G.F.; Atkins, K.E.; Funk, S.; Kucharski, A.J. Implication of Backward Contact Tracing in the Presence of Overdispersed Transmission in COVID-19 Outbreaks. *Wellcome Open Res.* **2021**, *5*, 1–17, doi:10.12688/WELLCOMEOPENRES.16344.3.
13. Ferrari, A.; Santus, E.; Cirillo, D.; Ponce-de-Leon, M.; Marino, N.; Ferretti, M.T.; Santuccione Chadha, A.; Mavridis, N.; Valencia, A. Simulating SARS-CoV-2 Epidemics by Region-Specific Variables and Modeling Contact Tracing App Containment. *Npj Digit. Med.* **2021**, *4*, doi:10.1038/s41746-020-00374-4.
14. Ferretti, L.; Wymant, C.; Kendall, M.; Zhao, L.; Nurtay, A.; Abeler-Dörner, L.; Parker, M.; Bonsall, D.; Fraser, C. Quantifying SARS-CoV-2 Transmission Suggests Epidemic Control with Digital Contact Tracing. *Science* **2020**, *368*, doi:10.1126/science.abb6936.
15. Gardner, B.J.; Kilpatrick, A.M. Contact Tracing Efficiency, Transmission Heterogeneity, and Accelerating COVID-19 Epidemics. *PLOS Comput. Biol.* **2021**, *17*, e1009122, doi:10.1371/journal.pcbi.1009122.
16. Ge, Y.; Chen, Z.; Handel, A.; Martinez, L.; Xiao, Q.; Li, C.; Chen, E.; Pan, J.; Li, Y.; Ling, F.; et al. The Impact of Social Distancing, Contact Tracing, and Case Isolation Interventions to Suppress the COVID-19 Epidemic: A Modeling Study. *Epidemics* **2021**, *36*, doi:10.1016/j.epidem.2021.100483.
17. Getz W.M.; Salter R.; Luisa Vissat L.; Horvitz N. A Versatile Web App for Identifying the Drivers of COVID-19 Epidemics. *J. Transl. Med.* **2021**, *19*, 109, doi:10.1186/s12967-021-02736-2.
18. Gill B.S.; Jayaraj V.J.; Singh S.; Ghazali S.M.; Cheong Y.L.; Md Iderus N.H.; Sundram B.M.; Aris T.B.; Mohd Ibrahim H.; Hong B.H.; et al. Modelling the Effectiveness of Epidemic Control Measures in Preventing the Transmission of COVID-19 in Malaysia. *Int. J. Environ. Res. Public. Health* **2020**, *17*, 1–13, doi:10.3390/ijerph17155509.
19. Giordano, G.; Blanchini, F.; Bruno, R.; Colaneri, P.; Di Filippo, A.; Di Matteo, A.; Colaneri, M. Modelling the COVID-19 Epidemic and Implementation of Population-Wide Interventions in Italy. *Nat. Med.* **2020**, *26*, 855–860, doi:10.1038/s41591-020-0883-7.
20. Grantz, K.H.; Lee, E.C.; D'Agostino McGowan, L.; Lee, K.H.; Metcalf, J.C.E.; Gurley, E.S.; Lessler, J. Maximizing and Evaluating the Impact of Test-Trace-Isolate Programs: A Modeling Study. *PLoS Med.* **2021**, *18*, doi:10.1371/journal.pmed.1003585.
21. Grimm, V.; Berger, U.; Bastiansen, F.; Eliassen, S.; Ginot, V.; Giske, J.; Goss-Custard, J.; Grand, T.; Heinz, S.K.; Huse, G.; et al. A Standard Protocol for Describing Individual-Based and Agent-Based Models. *Ecol. Model.* **2006**, *198*, 115–126, doi:10.1016/j.ecolmodel.2006.04.023.
22. Hellewell, J.; Abbott, S.; Gimma, A.; Bosse, N.I.; Jarvis, C.I.; Russell, T.W.; Munday, J.D.; Kucharski, A.J.; Edmunds, W.J.; Sun, F.; et al. Feasibility of Controlling COVID-19 Outbreaks by Isolation of Cases and Contacts. *Lancet Glob. Health* **2020**, *8*, e488–e496, doi:10.1016/S2214-109X(20)30074-7.
23. Hernandez-Orallo, E.; Manzoni, P.; Calafate, C.T.; Cano, J.-C. Evaluating How Smartphone Contact Tracing Technology Can Reduce the Spread of Infectious Diseases: The Case of COVID-19. *IEEE Access* **2020**, *8*, 99083–99097, doi:10.1109/ACCESS.2020.2998042.
24. Hinch, R.; Probert, W.J.M.; Nurtay, A.; Kendall, M.; Wymant, C.; Hall, M.; Lythgoe, K.; Bulas Cruz, A.; Zhao, L.; Stewart, A.; et al. OpenABM-Covid19-An Agent-Based Model for Non-Pharmaceutical Interventions against COVID-19 Including Contact Tracing. *PLoS Comput. Biol.* **2021**, *17*, doi:10.1371/journal.pcbi.1009146.

25. Hoops, S.; Chen, J.; Adiga, A.; Lewis, B.; Mortveit, H.; Baek, H.; Wilson, M.; Xie, D.; Swarup, S.; Venkatramanan, S.; et al. High Performance Agent-Based Modeling to Study Realistic Contact Tracing Protocols.; 2021; Vol. 2021-December.
26. Hornstein, A. Quarantine, Contact Tracing, and Testing: Implications of an Augmented SEIR Model. *BE J. Macroecon.* **2022**, *22*, 53–88, doi:10.1515/bejm-2020-0168.
27. Hu, Y.; Guo, J.; Li, G.; Lu, X.; Li, X.; Zhang, Y.; Cong, L.; Kang, Y.; Jia, X.; Shi, X.; et al. Role of Efficient Testing and Contact Tracing in Mitigating the COVID-19 Pandemic: A Network Modelling Study. *BMJ Open* **2021**, *11*, doi:10.1136/bmjopen-2020-045886.
28. Humphrey, L.; Thommes, E.W.; Fields, R.; Coudeville, L.; Hakim, N.; Chit, A.; Wu, J.; Cojocaru, M.G. Large-Scale Frequent Testing and Tracing to Supplement Control of Covid-19 and Vaccination Rollout Constrained by Supply. *Infect. Dis. Model.* **2021**, *6*, 955–974, doi:10.1016/j.idm.2021.06.008.
29. James, A.; Plank, M.J.; Hendy, S.; Binny, R.; Lustig, A.; Steyn, N.; Nesdale, A.; Verrall, A. Successful Contact Tracing Systems for COVID-19 Rely on Effective Quarantine and Isolation. *PLoS ONE* **2021**, *16*, doi:10.1371/journal.pone.0252499.
30. Kerr, C.C.; Mistry, D.; Stuart, R.M.; Rosenfeld, K.; Hart, G.R.; Núñez, R.C.; Cohen, J.A.; Selvaraj, P.; Abeysuriya, R.G.; Jastrzębski, M.; et al. Controlling COVID-19 via Test-Trace-Quarantine. *Nat. Commun.* **2021**, *12*, doi:10.1038/s41467-021-23276-9.
31. Khajanchi, S.; Sarkar, K.; Mondal, J.; Nisar, K.S.; Abdelwahab, S.F. Mathematical Modeling of the COVID-19 Pandemic with Intervention Strategies. *Results Phys.* **2021**, *25*, doi:10.1016/j.rinp.2021.104285.
32. Kim, Y.J.; Koo, P.-H. Effectiveness of Testing and Contact-Tracing to Counter COVID-19 Pandemic: Designed Experiments of Agent-Based Simulation. *Healthc. Switz.* **2021**, *9*, doi:10.3390/healthcare9060625.
33. Kretzschmar, M.E.; Rozhnova, G.; Bootsma, M.C.J.; van Boven, M.; van de Wijgert, J.H.H.M.; Bonten, M.J.M. Impact of Delays on Effectiveness of Contact Tracing Strategies for COVID-19: A Modelling Study. *Lancet Public Health* **2020**, *5*, e452–e459, doi:10.1016/S2468-2667(20)30157-2.
34. Kucharski, A.J.; Klepac, P.; Conlan, A.J.K.; Kissler, S.M.; Tang, M.L.; Fry, H.; Gog, J.R.; Edmunds, W.J.; Emery, J.C.; Medley, G.; et al. Effectiveness of Isolation, Testing, Contact Tracing, and Physical Distancing on Reducing Transmission of SARS-CoV-2 in Different Settings: A Mathematical Modelling Study. *Lancet Infect. Dis.* **2020**, *20*, 1151–1160, doi:10.1016/S1473-3099(20)30457-6.
35. Lanzarotti, E.; Santi, L.; Castro, R.; Roslan, F.; Groisman, L. A Multi-Aspect Agent-Based Model of COVID-19: Disease Dynamics, Contact Tracing Interventions and Shared Space-Driven Contagions.; 2021; Vol. 2021-December.
36. Maiorana, A.; Meneghelli, M.; Resnati, M. Effectiveness of Isolation Measures with App Support to Contain COVID-19 Epidemics: A Parametric Approach. *J. Math. Biol.* **2021**, *83*, doi:10.1007/s00285-021-01660-9.
37. Mancastroppa, M.; Castellano, C.; Vezzani, A.; Burioni, R. Stochastic Sampling Effects Favor Manual over Digital Contact Tracing. *Nat. Commun.* **2021**, *12*, doi:10.1038/s41467-021-22082-7.
38. McQuade, S.T.; Weightman, R.; Merrill, N.J.; Yadav, A.; Trélat, E.; Allred, S.R.; Piccoli, B. Control of COVID-19 Outbreak Using an Extended SEIR Model. *Math. Models Methods Appl. Sci.* **2021**, *31*, 2399–2424, doi:10.1142/S0218202521500512.
39. Mettler, S.K.; Park, J.; Özbek, O.; Mettler, L.K.; Ho, P.-H.; Rhim, H.C.; Maathuis, M.H. The Importance of Timely Contact Tracing — A Simulation Study. *Int. J. Infect. Dis.* **2021**, *108*, 309–319, doi:10.1016/j.ijid.2021.04.029.
40. Pollmann, T.R.; Schönert, S.; Müller, J.; Pollmann, J.; Resconi, E.; Wiesinger, C.; Haack, C.; Shtembari, L.; Turcati, A.; Neumair, B.; et al. The Impact of Digital Contact Tracing on the SARS-CoV-2 Pandemic—a Comprehensive Modelling Study. *EPJ Data Sci.* **2021**, *10*, doi:10.1140/epjds/s13688-021-00290-x.
41. Rajabi, A.; Mantzaris, A.V.; Mutlu, E.C.; Garibay, O.O. Investigating Dynamics of Covid-19 Spread and Containment with Agent-Based Modeling. *Appl. Sci. Switz.* **2021**, *11*, doi:10.3390/app11125367.
42. Ramos, A.M.; Ferrández, M.R.; Vela-Pérez, M.; Kubik, A.B.; Ivorra, B. A Simple but Complex Enough θ-SIR Type Model to Be Used with COVID-19 Real Data. Application to the Case of Italy. *Phys. Nonlinear Phenom.* **2021**, *421*, doi:10.1016/j.physd.2020.132839.
43. Rusu, A.C.; Emonet, R.; Farrahi, K. Modelling Digital and Manual Contact Tracing for COVID-19. Are Low Uptakes and Missed Contacts Deal-Breakers? *PLoS ONE* **2021**, *16*, doi:10.1371/journal.pone.0259969.
44. Ryu, H.; Abulali, A.; Lee, S. Assessing the Effectiveness of Isolation and Contact-Tracing Interventions for Early Transmission Dynamics of COVID-19 in South Korea. *IEEE Access* **2021**, *9*, 41456–41467, doi:10.1109/ACCESS.2021.3064371.
45. Sasmita, N.R.; Ikhwan, M.; Suyanto, S.; Chongsuvivatwong, V. Optimal Control on a Mathematical Model to Pattern the Progression of Coronavirus Disease 2019 (COVID-19) in Indonesia. *Glob. Health Res. Policy* **2020**, *5*, doi:10.1186/s41256-020-00163-2.
46. Scarabel, F.; Pellis, L.; Ogden, N.H.; Wu, J. A Renewal Equation Model to Assess Roles and Limitations of Contact Tracing for Disease Outbreak Control. *R. Soc. Open Sci.* **2021**, *8*, doi:10.1098/rsos.202091.
47. Shayak B.; Sharma M.M. Contact Tracing Can Explain Counter-Intuitive COVID-19 Trajectories, Mitigate Disease Transmission and Provide an Early Warning Indicator - A Mathematical Modeling Study. *medRxiv* **2021**, doi:10.1101/2021.09.27.21264174.
48. Soldano, G.J.; Fraire, J.A.; Finochietto, J.M.; Quiroga, R. COVID-19 Mitigation by Digital Contact Tracing and Contact Prevention (App-Based Social Exposure Warnings). *Sci. Rep.* **2021**, *11*, doi:10.1038/s41598-021-93538-5.
49. Sturniolo, S.; Waites, W.; Colbourn, T.; Manheim, D.; Panovska-Griffiths, J. Testing, Tracing and Isolation in Compartmental Models. *PLoS Comput. Biol.* **2021**, *17*, doi:10.1371/journal.pcbi.1008633.

50. Tatapudi, H.; Das, R.; Das, T.K. Impact Assessment of Full and Partial Stay-at-Home Orders, Face Mask Usage, and Contact Tracing: An Agent-Based Simulation Study of COVID-19 for an Urban Region. *Glob. Epidemiol.* **2020**, *2*, doi:10.1016/j.gloepi.2020.100036.
51. Traoré, A.; Konané, F.V. Modeling the Effects of Contact Tracing on COVID-19 Transmission. *Adv. Differ. Equ.* **2020**, *2020*, doi:10.1186/s13662-020-02972-8.
52. Wang T.; Wu Y.; Lau J.Y.-N.; Yu Y.; Liu L.; Li J.; Zhang K.; Tong W.; Jiang B. A Four-Compartment Model for the COVID-19 Infection-Implications on Infection Kinetics, Control Measures, and Lockdown Exit Strategies. *Precis. Clin. Med.* **2020**, *3*, 104–112, doi:10.1093/pcmedi/pbaa018.
53. Grimm, V.; Mengel, F.; Schmidt, M. Extensions of the SEIR Model for the Analysis of Tailored Social Distancing and Tracing Approaches to Cope with COVID-19. *Sci. Rep.* **2021**, *11*, doi:10.1038/s41598-021-83540-2.
